# Estradiol increases cortical and trabecular bone accrual and bone strength in an adolescent male-to-female mouse model of gender-affirming hormone therapy

**DOI:** 10.1038/s41413-023-00308-2

**Published:** 2024-01-11

**Authors:** Tian Nie, Varun S. Venkatesh, Suzanne Golub, Kathryn S. Stok, Haniyeh Hemmatian, Reena Desai, David J. Handelsman, Jeffrey D. Zajac, Mathis Grossmann, Rachel A. Davey

**Affiliations:** 1 Department of Medicine, Austin Health, University of Melbourne, Heidelberg, VIC 3084 Australia; 2 Department of Biomedical Engineering, University of Melbourne, Parkville, VIC 3010 Australia; 3 ANZAC Research Institute, University of Sydney and Andrology, Concord Repatriation General Hospital, Concord, NSW 2137 Australia

**Keywords:** Osteoporosis, Bone, Bone quality and biomechanics

## Abstract

The effects of gender-affirming hormone therapy on the skeletal integrity and fracture risk in transitioning adolescent trans girls are unknown. To address this knowledge gap, we developed a mouse model to simulate male-to-female transition in human adolescents in whom puberty is first arrested by using gonadotrophin-releasing hormone analogs with subsequent estradiol treatment. Puberty was suppressed by orchidectomy in male mice at 5 weeks of age. At 3 weeks post-surgery, male-to-female mice were treated with a high dose of estradiol (~0.85 mg) by intraperitoneal silastic implantation for 12 weeks. Controls included intact and orchidectomized males at 3 weeks post-surgery, vehicle-treated intact males, intact females and orchidectomized males at 12 weeks post-treatment. Compared to male controls, orchidectomized males exhibited decreased peak bone mass accrual and a decreased maximal force the bone could withstand prior to fracture. Estradiol treatment in orchidectomized male-to-female mice compared to mice in all control groups was associated with an increased cortical thickness in the mid-diaphysis, while the periosteal circumference increased to a level that was intermediate between intact male and female controls, resulting in increased maximal force and stiffness. In trabecular bone, estradiol treatment increased newly formed trabeculae arising from the growth plate as well as mineralizing surface/bone surface and bone formation rate, consistent with the anabolic action of estradiol on osteoblast proliferation. These data support the concept that skeletal integrity can be preserved and that long-term fractures may be prevented in trans girls treated with GnRHa and a sufficiently high dose of GAHT. Further study is needed to identify an optimal dose of estradiol that protects the bone without adverse side effects.

## Introduction

Transgender (or trans) people have a gender identity that differs from their birth sex,^1^ while cisgender (e.g., cis female, cis male) individuals have a gender identity that aligns with their birth sex. Gender-affirming hormone therapy (GAHT) is commonly used to align a trans person’s physical characteristics with their gender identity to relieve dysphoria, depression, and suicidal ideation. Trans women (assigned male at birth but identify as female/feminine) receive estradiol (with or without anti-androgens). To avoid the development of undesired pubertal characteristics of their natal sex prior to commencing GAHT, trans adolescents can consider puberty suppression as part of their medical therapy in the context of a multidisciplinary assessment by treatment with gonadotrophin-releasing hormone analogs (GnRHa). GnRHa decrease circulating concentrations of sex steroids by decreasing the secretion of luteinizing hormone (LH) and follicle-stimulating hormone (FSH), maintaining circulating sex steroids at prepubertal levels. Pubertal suppression is commonly followed by GAHT to align the postpubertal characteristics of trans adolescents’ experienced gender.

It is well established that sex steroids are crucial for peak bone mass accrual and growth during puberty and for bone maintenance and strength in adulthood in both cis males and cis females.^2^ It is not surprising, therefore, that pubertal suppression with GnRHa therapy prevents the attainment of peak bone mass evidenced by decreases in areal bone mineral density (aBMD), volumetric BMD (vBMD) and bone modeling in both trans girls and trans boys compared to age-matched cis controls of the natal gender.^3–7^ Commencement of GAHT in trans girls and trans boys can partially reverse the deficit in BMD, but current treatment regimens may not be sufficient to restore BMD to levels of age-matched cis controls undergoing spontaneous puberty, potentially placing these trans individuals at greater risk of fracture later in life.^3–5,7^ Of particular concern, in trans girls, it has been reported that the baseline lumbar and femoral neck bone mineral apparent density (BMAD) Z scores are below the population mean and remain below zero following three years of combined GnRHa therapy and GAHT.^7^ Likewise, in a more recent cohort study, even after long-term use of GAHT (median 11.6 years), in trans girls previously receiving GnRHa therapy to suppress puberty, the lumbar spine Z scores did not catch up with pretreatment levels.^8^ GAHT is typically administered life long, yet to date, there is very limited information regarding the long-term effects of GnRHa/GAHT in trans adolescents on their skeletal integrity, and there are no data relating to their fracture risk.

In humans and mice of both sexes, estradiol is the dominant sex steroid regulating bone resorption, with both testosterone and estradiol being important for bone formation.^9–12^ The concentrations of estradiol within bone can be derived passively from the circulating blood stream or actively from the local conversion of testosterone within bone by aromatase expressed primarily by osteoblasts and at lower levels in osteocytes and chondrocytes.^13,14^ The relative contributions of estradiol derived from the circulation versus its local synthesis within bone to skeletal integrity are not known. The importance of aromatase activity within bone in preserving skeletal integrity has been demonstrated in male mice overexpressing aromatase specifically in osteoblasts in which BMD was markedly increased compared to controls.^15^ While no effect of aromatase overexpression was observed in females, this was attributed to their low concentrations of circulating testosterone as a substrate for aromatization to estradiol. Subsequent treatment with exogenous testosterone increased BMD, reinforcing the notion that local aromatization of testosterone to estradiol may be essential for maintaining skeletal integrity.^15^ These data suggest that in trans girls with low serum testosterone concentrations treated with GnRHa and GAHT,^16^ the major determinant of bone integrity could be the local concentrations of estradiol within bone derived from exogenous circulating estradiol.

Interpreting the effects of puberty suppression and GAHT on skeletal integrity and fracture risk in adolescent trans people is challenging because existing studies have included differing treatment regimens, relied on inaccurate sex steroid immunoassays and lacked appropriate control groups. An additional limitation that confounds accurate interpretation is the cross-sectional studies reporting that trans girls have a lower BMD than cis boys even prior to the commencement of GAHT.^7^ As such, differences in BMD in trans girls cannot be ascribed solely to the effects of GAHT. Since withholding GAHT is considered unethical, randomized placebo-controlled, long-term clinical trials necessary for determining fracture risk in trans people are unlikely to be conducted, and crucially, direct measures of bone strength to precisely determine fracture risk are not possible in humans. To address some of these limitations, we established a mouse model of male-to-female transition in adolescence mimicking the clinical approach in trans girls when puberty is first arrested, with subsequent estradiol treatment. We hypothesized that the skeletal integrity and bone strength of pubertally suppressed male-to-female mice will be maintained with GAHT administration if the local concentrations of estradiol within bone are sufficient. Although two recent preclinical reports have focused on female-to-male transition,^17,18^ to our knowledge, this is the first report of a preclinical model of the effects of male-to-female transition during puberty on skeletal integrity.

## Results

### Circulating estradiol concentrations are elevated and testosterone concentrations are decreased in orchidectomized (Orx) male-to-female mice administered estradiol

**Fig. 1:**
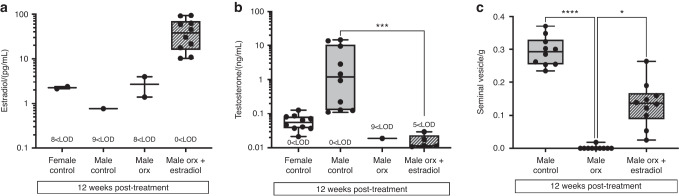
Serum estradiol concentrations are elevated in Orx male-to-female mice administered estradiol by silastic implantation. Serum (**a**) estradiol (pg/mL) and (**b**) testosterone (ng/mL) in intact female, intact male, and male Orx controls and in male Orx mice administered estradiol (+ estradiol). The text below each experimental group indicates the number of samples below the limit of detection (LOD) of the LC/MS assay. **c** Seminal vesicle mass (mg) in male controls, Orx males and male Orx mice administered estradiol (+ estradiol) at 3 weeks post-surgery (8 weeks of age) for 12 weeks (20 weeks of age), *n* = 10/group. Serum hormone concentrations are plotted on a logarithmic scale. Shown are individual values, medians, interquartile ranges, and minimum and maximum values. *P < 0.05, **P < 0.01, ***P < 0.000 5, ****P < 0.000 1

At 12 weeks post-treatment, serum estradiol concentrations did not differ between female, male, and Orx male controls, with most serum samples being below the limit of detection (LOD) of the assay. Compared to all controls, Orx male-to-female mice treated with estradiol exhibited ∼30-fold increased serum estradiol concentrations [median (IQR) 37.9 (15.9, 69.8) pg/mL], reaching concentrations of ∼2.7-fold higher than the peak concentration of estradiol that occurs in the proestrus stage of the estrus cycle in C57BL/6 female mice [median (IQR) 13.8 (9.7, 30) pg/mL] measured with the same LCMS method^19^ (Fig. 1a). Serum testosterone was decreased in Orx male-to-females compared to intact male controls with complete regression of the seminal vesicles observed in all but one of these mice (Fig. 1b, c).

### Pubertal suppression reduces peak cortical bone accrual, which is increased by estradiol treatment in Orx male-to-female mice, resulting in increased bone strength

**Fig. 2:**
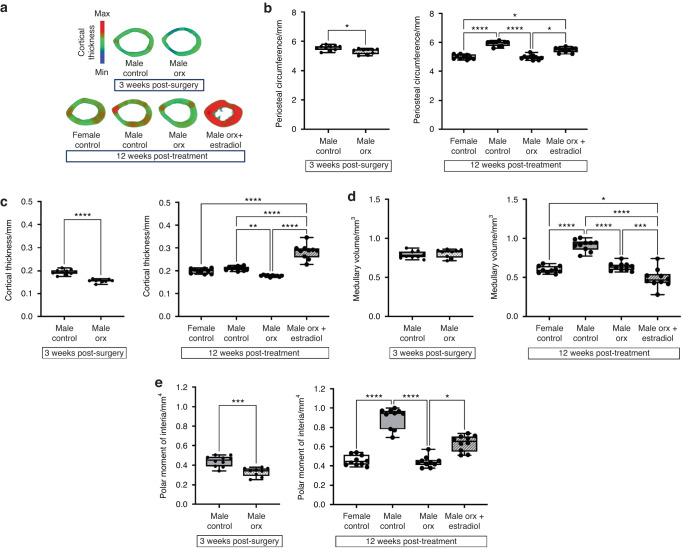
Pubertal suppression decreases peak cortical bone accrual, which is increased by subsequent estradiol treatment in Orx male-to-female mice. **a** Representative μCT images showing the femoral cross-section averaged over the imaging depth of 0.68 mm at the mid-diaphysis. Scale on left indicates cortical thickness. **b** Periosteal circumference (mm), (**c**) cortical thickness (mm), (**d**) medullary volume (mm^3^), and (**e**) polar moment of inertia (mm^4^) in male controls and Orx males 3 weeks post-surgery (8 weeks of age) and in female controls, male controls, Orx males and Orx males administered estradiol (male Orx + estradiol) at 3 weeks post-surgery (8 weeks of age) for 12 weeks (20 weeks of age) (*n* = 10/group). *P < 0.05, **P < 0.005, ***P < 0.001, ****P < 0.000 1. Shown are individual values, medians, interquartile ranges, and minimum and maximum values

The femoral length did not differ between the experimental groups at their respective ages (Table 1). Growth plate thickness was decreased, and periosteal circumference and medullary volume were increased in intact male controls compared to females at 12 weeks post-treatment, consistent with the sexual dimorphism of bone (Table 1, Fig. 2a–e).^20^ Orchidectomy led to a reduction in the periosteal circumference and cortical thickness (Ct.Th) at the mid-diaphysis at 3 weeks post-surgery and 12 weeks post-vehicle treatment compared to intact male but not intact female controls (Fig. 2b, c). A decrease in medullary volume in Orx males compared to intact male controls was evident by 15 weeks post-surgery (12 weeks post-vehicle treatment) (Fig. 2d). The reduction in cortical bone in Orx mice was reflected by a decrease in the polar moment of inertia (pMOI) at both 3 weeks post-surgery and 12 weeks post-vehicle treatment, a calculated geometric property of a cross-section of bone that expresses its inherent resistance to rotational torque,^21^ suggesting a decrease in torsional bone strength (Fig. 2e). Estradiol treatment of male-to-female Orx males at 3 weeks post-surgery increased the growth plate thickness and periosteal circumference to levels that were intermediate between intact male and female controls (Table 1, Fig. 2b). Ct.Th was increased in estradiol-treated male-to-female Orx males compared to intact female, intact male and Orx male controls (Fig. 2c), which was accompanied by a concomitant decrease in medullary volume (Fig. 2d). The increase in cortical bone in estradiol-treated Orx males compared to female and male Orx controls was associated with an increase in the pMOI indicative of increased strength, but pMOI in estradiol-treated Orx males did not differ from male controls (Fig. 2e).

**Table 1:** Femur length and body weight in sham-operated 8-week-old male mice (Male Control) or orchidectomized male (Male Orx) at 5 weeks of age, in Female Control, Male Control and Male Orx mice administered vehicle, and in Male Orx mice administered estradiol (Male Orx + estradiol) at 3 weeks post-surgery (8 weeks of age) for 12 weeks (20 weeks of age)

	3 weeks post-surgery		12 weeks post-treatment			
Parameter	Male control	Male Orx	Female control	Male control	Male Orx	Male Orx + estradiol
Number of samples	10	9	10	10	10	10
Body weight/g	22.9 ± 0.5	21.4 ± 0.2^#^	21.3 ± 0.4	29.7 ± 0.8^****^	27.2 ± 0.6^****,#^	28.7 ± 0.6^****^
Femur length/mm	15.0 ± 0.1	14.8 ± 0.1	15.3 ± 0.1	15.7 ± 0.1	15.5 ± 0.1	15.8 ± 0.1
Growth plate thickness/μm	66.5 ± 3.2	70.1 ± 2.5	52.5 ± 2.0	34.9 ± 2.0^***^	52.8 ± 3.2^###^	42.0 ± 3.5^$^

Values are the mean ± SE, ***P = 0.000 5, ****P < 0.000 1 versus female control within age group, ^#^*P* < 0.05, ^###^*P* < 0.000 5 versus male control, ^$^*P* < 0.05 versus Male Orx

**Fig. 3:**
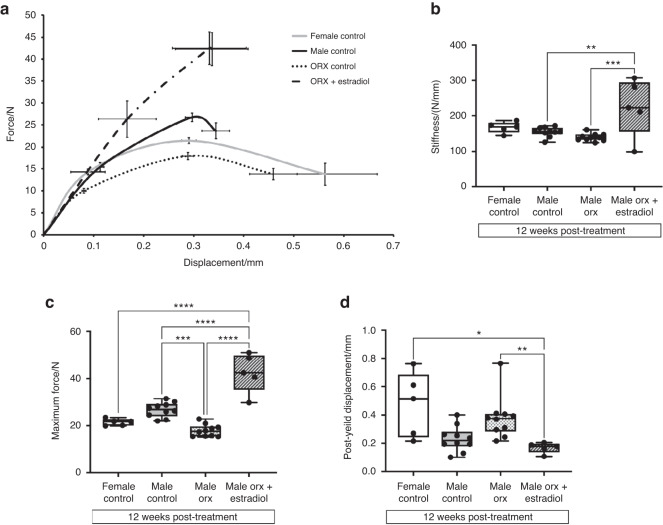
Estradiol treatment in Orx male-to-female mice increases whole-bone strength. **a** Mean force (N) vs. displacement (mm) for each group with error bars representing SEM from which the following parameters (**b**–**d**) were derived: (**b**) stiffness (N/mm), (**c**) maximum force (N), (**d**) postyield displacement (mm) in female controls (*n* = 5), male controls (*n* = 10), Orx males (*n* = 10) and Orx males administered estradiol ( + estradiol) (*n* = 5) at 3 weeks post-surgery (8 weeks of age) for 12 weeks (20 weeks of age). *P < 0.05, **P < 0.01, ***P < 0.000 5, ****P < 0.000 1. Shown are individual values, medians, interquartile ranges, and minimum and maximum values

To determine if the increase in cortical bone size and thickness in Orx male-to-female mice following estradiol treatment increased bone strength, 3-point bending analyses at the mid-diaphysis were performed. The mean force versus displacement graph depicted in Fig. 3a summarizes the whole-bone strength for each of the four experimental groups at 12 weeks post-treatment (20 weeks of age), from which the following parameters were derived: stiffness, maximum force, postyield displacement and work to fracture (area under the curve). The maximal force was decreased in Orx males compared to intact male controls, while stiffness, postyield displacement and work-to-fracture were unaffected (Fig. 3b–d). Estradiol treatment of Orx male-to-female mice markedly increased the stiffness and maximal force that the mid-diaphysis could withstand prior to fracture (Fig. 3b, c). Postyield displacement was decreased in Orx male-to-females treated with estradiol compared to Orx male controls (Fig. 3d).

### Estradiol treatment of Orx male-to-female mice markedly increases trabecular bone volume due to increased bone formation

**Fig. 4:**
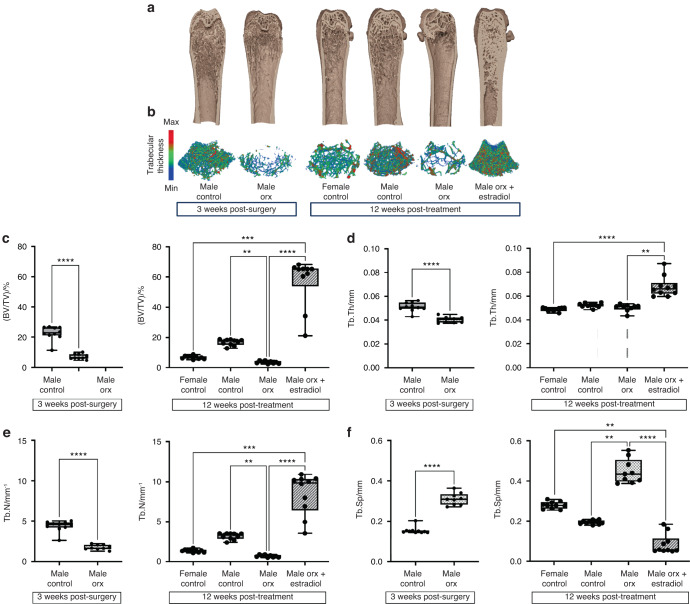
Pubertal suppression decreases peak trabecular bone accrual, which is markedly increased with subsequent estradiol treatment in Orx male-to-female mice. Representative 3D reconstructed μCT images showing (**a**) the distal femur and (**b**) trabecular bone in the distal femoral metaphysis. Scale on left indicates trabecular thickness. **c** Trabecular bone volume as a % of bone volume to tissue volume ((BV/TV)/%), (**d**) trabecular thickness (Tb.Th/mm), (**e**) trabecular number (Tb.N/mm^−1^) and (**f**) trabecular separation (Tb.Sp/mm) in male controls (*n* = 10) and Orx males (*n* = 10) 3 weeks post-surgery (8 weeks of age) and in female controls (*n* = 10), male controls (*n* = 10), Orx males (*n* = 9) and Orx males administered estradiol (male Orx + estradiol) (*n* = 10) at 3 weeks post-surgery (8 weeks of age) for 12 weeks (20 weeks of age). **P < 0.005, ***P < 0.000 5, ****P < 0.000 1. Shown are individual values, medians, interquartile ranges, and minimum and maximum values

**Fig. 5:**
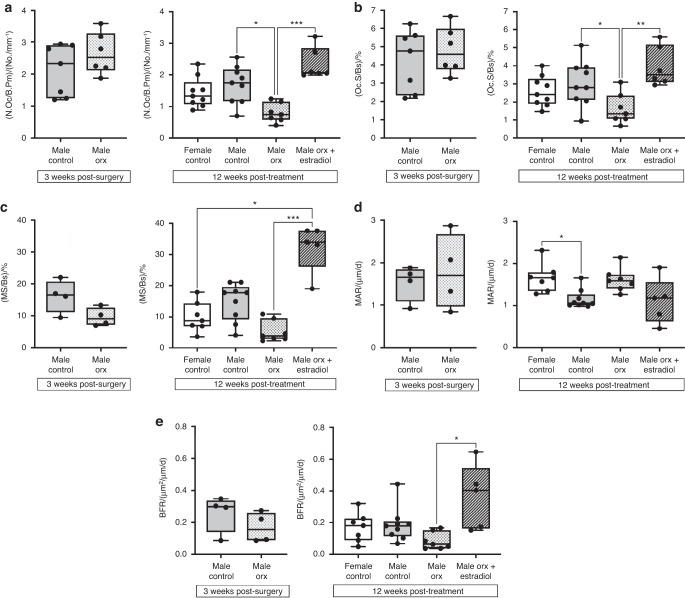
Estradiol treatment in Orx male-to-female mice increases trabecular bone accrual by stimulating bone formation. **a** Osteoclast number/bone perimeter [(N.Oc./B.Pm)/(No./mm^−1^)], (**b**) osteoclast surface/bone surface ((Oc.S/BS)/%), (**c**) mineralizing surface/bone surface ((MS/BS)/%), (**d**) mineral apposition rate (MAR/(μm/d)), (**e**) bone formation rate (BFR/(μm^2^/μm/d)) in the distal femoral metaphysis of male controls (*n* = 4 for (**a**, **b**), *n* = 7 for (**c**–**e**)) and Orx males (*n* = 4 for (**a**, **b**), *n* = 6 for (**c**–**e**)) 3 weeks post-surgery (8 weeks of age) and in female controls (*n* = 7–8), male controls (*n* = 9), Orx males (*n* = 7) and Orx males administered estradiol (male Orx + estradiol) (*n* = 5–6) at 3 weeks post-surgery (8 weeks of age) for 12 weeks (20 weeks of age). *P < 0.05, **P < 0.005, ***P < 0.000 5. Shown are individual values, medians, interquartile ranges, and minimum and maximum values

Compared to intact male controls, Orx mice exhibited a decrease in trabecular bone volume per tissue volume (BV/TV) at 3 weeks post-orchidectomy due to a reduction in trabecular number (Tb.N) and trabecular thickness (Tb.Th), while trabecular separation (Tb.Sp) was increased (Fig. 4a–f). At this time point post-orchidectomy, the bone remodeling parameters, namely, osteoclast number per bone perimeter (N.Oc/B.Pm), osteoclast surface per bone surface (Oc.S/BS), mineralizing surface (MS/BS), mineral apposition rate (MAR) and bone formation rate (BFR), did not differ between Orx and intact males (Fig. 5a–e). The expression levels of osteoclast genes (*Acp5*, *Ctsk*) and osteoblast genes (*Col1a1*, *Bglap*) were unchanged in whole bone between Orx males and intact males, except for *Dc-stamp*, which was downregulated in Orx males compared to male controls (Fig. S1A–E). Orchidectomy had no effect on growth plate thickness at 3 weeks post-surgery, but by 20 weeks post-vehicle treatment, the growth plate thickness was increased to female control levels (Table 1).

**Fig. 6:**
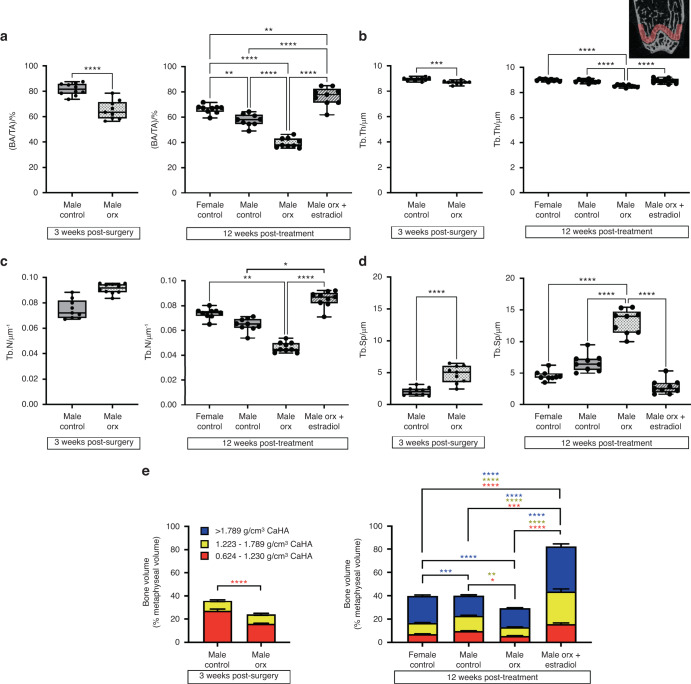
Estradiol treatment in Orx male-to-female mice increases trabecular bone arising from the growth plate. **a** Bone area as a percentage of total area ((BA/TA)/%), (**b**) trabecular thickness (Tb.Th/μm), (**c**) trabecular number (Tb.N/mm^−1^) and (**d**) trabecular separation (Tb.Sp/μm) in the metaphyseal primary spongiosa of male controls and (**e**) trabecular and cortical bone volume as a percentage of total metaphyseal volume measured at low-, mid- and high-density thresholds in male controls (*n* = 10) and Orx males (*n* = 10) 3 weeks post-surgery (8 weeks of age) and in female controls (*n* = 10), male controls (*n* = 10), Orx males (*n* = 9) and Orx males administered estradiol (male Orx + estradiol) (*n* = 10) at 3 weeks post-surgery (8 weeks of age) for 12 weeks (20 weeks of age). For (**a**–**d**), the inset depicts the region of interest for primary spongiosa measurements extending 265 μm from the growth plate. *P < 0.01, **P < 0.005, ***P < 0.001, ****P < 0.000 1. For (**e**), the colors used for *P* values match the density level of bone analyzed; data are the mean ± standard error; *P < 0.05, **P < 0.005, ***P < 0.000 5, ****P < 0.000 1. Shown are individual values, medians, interquartile ranges, and minimum and maximum values

The decreased BV/TV in Orx males persisted to adulthood at 12 weeks post-vehicle treatment (20 weeks of age) (Fig. 4c). Estradiol treatment of Orx male-to-female mice led to a marked increase in BV/TV of 9.5- and 18-fold compared to intact female and male Orx controls, respectively, primarily due to an increase in Tb.N, resulting in a decrease in Tb.Sp, while Tb.Th was increased by 1.3-fold (Fig. 5c–f). The dramatic increase in BV/TV following estradiol treatment in Orx male-to-female mice compared to male Orx controls was associated with an increase in MS/BS and BFR, while MAR was unaffected (Fig. 5c–e). N.Oc/B.Pm and Oc.S/BS were also increased in estradiol-treated male-to-female Orx mice compared to both intact and Orx male controls (Fig. 5a, b). Similarly, the expression of genes involved in bone remodeling were markedly upregulated in whole bone in estradiol-treated male-to-female Orx mice compared to intact female, intact male and Orx male controls (Fig. S1A–E). The marked increase in trabecular bone accrual following estradiol treatment in Orx male-to-female mice compared to male Orx controls was associated with an increase in newly growing trabecular bone arising from the growth plate, as evidenced by increases in the bone area/total area (BA/TA), Tb.Th. and Tb.N., and a decrease in Tb.Sep. in the primary spongiosa of the distal metaphysis (Fig. 6a–d).

To determine whether orchidectomy in pubertal male mice and the treatment of these mice 3 weeks post-orchidectomy with estradiol altered the mineral content of the cortical and trabecular bone tissue, multilevel Otsu thresholding was performed in the metaphyseal region. In pubertal mice, the metaphyseal region consisted of only low- and mid-density bone consistent with peak bone mass accrual occurring at 16 weeks of age in C57Bl/6 mice.^22^ Orchidectomy decreased the proportion of low- but not mid-density bone compared to intact male controls. At 12 weeks post-vehicle treatment (15 weeks post-surgery), decreases in both low- and mid-density bone were observed in Orx males, while the proportion of high-density bone was retained at similar levels to that observed in intact males. The striking increase in both cortical and trabecular bone that occurred following estradiol treatment in Orx male-to-female mice compared to intact female, intact male and Orx male controls comprised increases in all densities of metaphyseal bone (Fig. 6e).

## Discussion

Current clinical evidence shows that pubertal suppression by treatment with GnRHa prevents the attainment of peak bone mass accrual in both trans boys and trans girls, which is only partially restored in trans girls following commencement of GAHT at a later age.^3,4,7^ It is unclear whether this deficit in peak bone mass in trans girls treated with GnRHa and GAHT persists until adulthood and whether it increases the risk of fracture. This is of particular importance, as an increase in fracture risk has been reported in two cohorts of trans women, compared to cis men, who did not undergo pubertal suppression but were treated with GAHT alone or in combination with gender-confirming surgery commencing in adulthood even after peak bone mass had been reached.^23,24^ Hence, it is possible that bone health in trans girls may be further compromised by current clinical approaches (e.g., GnRHa therapy), which impede the acquisition of peak bone mass. Importantly, these previous studies compared fracture risk in trans women compared to cis male controls but not to cis females. To address this important knowledge gap while overcoming the difficulties inherent in clinical trials in this population, we developed a mouse model of male-to-female transition in adolescence to investigate the effects of pubertal suppression and subsequent GAHT with estradiol on peak bone mass accrual, microstructure, and strength. Pubertal mice were orchidectomized at 5 weeks of age and administered estradiol at 3 weeks post-surgery for a period of 12 weeks to replicate pubertal suppression in early adolescence in trans girls with GAHT commencing sometime later. Skeletal integrity was assessed in adulthood in male-to-female mice at 20 weeks of age at a time when peak bone mass is expected to have been achieved (16 weeks in C57Bl/6 mice)^22^ and at an age that equates to 26-27 years in trans women.^25^ A strength of this study was the inclusion of multiple control groups, including both intact male and female controls, to allow comparisons between mice of the same birth sex as well as the desired gender following transitioning.

Puberty suppression by orchidectomy in pubertal male mice compared to male controls decreased peak bone mass accrual, with failure of cortical bone accumulation at both the periosteal and endosteal surfaces and trabecular bone in the metaphysis evident within the first 3 weeks post-surgery. The decrease in BV/TV in the metaphysis was associated with a reduction in the newly formed primary spongiosa arising from the growth plate characterized by a decrease in both the trabecular thickness and number, which is in accordance with trabecular bone accrual during puberty in males being dependent on androgen receptor (AR) activation in proliferating osteoblasts.^26^ The decrease in pubertal trabecular and cortical bone accrual in the metaphysis of Orx males was associated with bone of low mineral density, while the amount of mid-density bone accumulated prior to puberty was maintained. Oc.S/BS and N.Oc/B.Pm were decreased in Orx males at 12 weeks post-vehicle treatment compared to male controls, indicating that resorption was suppressed in trabecular bone. Consistent with this finding, the gene expression levels of *Dcstamp*, one of the key mediators of osteoclastogenesis, and *Ctsk*, a protease that degrades bone matrix, were decreased in whole bone of Orx males at 3 weeks post-surgery. In contrast, the expression of *Acp5*, a metalloenzyme in the acid phosphatase category, was unchanged. Similarly, mineral apposition, bone formation rates and expression of the osteoblast genes *Col1a1* and *Bglap* were unaffected. The expression of all remodeling genes analyzed was low in all control groups at 12 weeks post-treatment (20 weeks of age), consistent with the age-related decline in bone remodeling.^27^ These data are consistent with previous reports of decreased cortical and trabecular bone acquisition in male rats and mice that were orchidectomized pre- or early puberty^28,29^ or administered high doses of GnRHa^30^ and assessed at 4 and 8 weeks post-surgery or 16 weeks post-GnRHa treatment, respectively. The results reported in the current and previous studies showing a decreased periosteal circumference and marked reduction in trabecular bone accrual following pubertal suppression by orchidectomy in male mice are consistent with AR signaling being the dominant pathway for periosteal and trabecular apposition of bone during puberty in male mice,^20,29,31,32^ while the decrease in cortical thickness is consistent with the increased endosteal erosion surface reported in prepubertal male mice Orx at 3 weeks of age.^31^

As expected, the failure to reach peak bone mass in Orx male-to-female mice at 20 weeks of age (12 weeks post-vehicle treatment) led to a reduction in bone strength as measured by pMOI and the maximal force the bone could withstand prior to fracture compared to intact male controls. Notably, however, although peak bone mass and bone strength were decreased in Orx male mice compared to male controls, there was no difference in any of the trabecular, cortical, mechanical or bone remodeling parameters compared to those of age-matched female controls at 20 weeks of age except for a reduction in trabecular BA/TA of the primary spongiosa. These data highlight the importance of careful consideration of control groups for clinical and preclinical studies of gender transition. While most clinical studies compare parameters between trans individuals and cis controls of the same birth sex, based on these data where the removal of endogenous sex steroids in Orx males led to female-like bones with the same strength as female controls, one might argue that comparisons between trans individuals and cis controls of the desired gender may be more appropriate, especially when assessing fracture risk.

The failure to reach peak bone mass in our early pubertal Orx mouse model compared to male but not female controls correlates well with longitudinal studies of transgender girls undergoing pubertal suppression with GnRHa therapy. In early pubertal trans girls defined by young bone age (<15 years)^3^ or Tanner stage 2 or 3,^7^ GnRHa treatment for a median of 1 or 2 years decreased BMAD Z scores of the lumbar spine, which was accompanied by a reduction in serum markers of bone remodeling. Similar observations were reported by Joseph et al. whereby GnRHa therapy in trans girls (average age of 13 years) resulted in a progressive decline in BMD and BMAD Z scores over 3 years, with the most rapid decrease occurring within the first year.^33^ It is important to note, however, that Z scores compare BMD to the average values of the same birth sex and age, while absolute BMD and BMAD of the femoral neck and lumber have been reported to be unchanged in each of these studies treating trans girls in early puberty with GnRHa for between 1 and 3 years, indicating an impairment to bone mass accrual rather than a loss of bone.^3,7,33^

To test whether GAHT in our male-to-female mouse model could restore bone accrual and strength in the pubertally suppressed male-to-female mice, we administered a high physiological dose of estradiol that was ∼2.7-fold higher than the peak estradiol levels that occur in the proestrus stage of the estrus cycle in female mice^19^ at 3 weeks post-orchidectomy for 12 weeks. As expected, serum concentrations of testosterone were decreased in Orx male-to-female mice, modeling serum concentrations observed in trans girls on GAHT.^16^ Estradiol treatment in male-to-female mice compared to all control mice increased the cortical thickness at the mid-diaphysis, consistent with the actions of high-dose estradiol to stimulate the endocortical deposition of bone previously reported in young ovariectomized female mice.^34^ Of interest, a bone size similar to that observed at 3 weeks post-orchidectomy was maintained with estradiol treatment of Orx male-to-female mice for 12 weeks, such that the periosteal circumference was at an intermediate level between male sham and Orx controls. These data are consistent with the hypothesis proposed by Venken et al. ^29^ that signaling via both the AR and estrogen receptor (ER) is needed for the peak stimulation of periosteal growth during puberty in male mice. The stimulatory effects of estradiol on periosteal expansion have also been reported in a case study of a 17-year-old boy with aromatase deficiency in whom treatment with estradiol for 3 years partially restored the bone size to levels observed in control males.^35^

Since disorders of overly dense bone, such as osteopetrosis, can lead to increased fragility, 3-point bending analyses were performed on the femur to determine the effect of the marked increase in cortical bone accrual at the mid-diaphysis following estradiol treatment in the male-to-female mice. Three-point bending analysis confirmed that the larger cross-sectional area and thicker cortices at the mid-diaphysis of the estradiol-treated Orx male-to-female mice made the bone stiffer and stronger and able to withstand a higher maximal force compared to all control groups. In addition, bone stiffness was higher while postyield displacement was lower in estradiol-treated Orx male-to-female mice compared to male Orx controls, which may be attributed to a decrease in the ratio of collagen to bone mineral content.^36^ Bending stress could be measured in future experiments to account for the increased cross-sectional area; however, this was not possible in the current study due to technical limitations. We also acknowledge that it remains theoretically possible that bone strength might be compromised at other sites in estradiol-treated Orx male-to-female mice, a possibility to be addressed by future work.

Estradiol treatment in pubertal Orx male-to-female mice markedly increased all the mineral density ranges (low, mid, upper) of trabecular and cortical bone in the distal femoral metaphysis compared to all control groups. The 18-fold increase in trabecular bone accrual was primarily driven by a concomitant 14-fold increase in trabecular number associated with an increase in the number of trabeculae arising from the growth plate in the primary spongiosa compared to Orx male controls. This was accompanied by an increase in the number of mineralizing surfaces and the bone formation rate within the secondary spongiosa of the metaphysis, while the mineral apposition rate was unaffected. The anabolic effect of estradiol treatment in male-to-female mice was remarkable given the well-established ability of estradiol to inhibit bone resorption; however, these findings are consistent with those of previous reports that estradiol increases osteoblast proliferation and inhibits apoptosis in vitro.^37–39^ Similarly, high doses of estradiol (500 μg once per week for 4 weeks) in young female ovariectomized mice compared to intact female and ovariectomized female controls have been shown to increase BV/TV by increasing the number of bone forming surfaces in vivo.^34^ The anabolic action of estradiol to increase bone accrual in Orx male-to-female mice was accompanied by an increase in the markers of bone resorption (Oc.S/BS, N.Oc/B.Pm and Dc-stamp, Acp5 and Ctsk gene expression). Considering the well-documented antiresorptive and proapoptotic actions of estradiol on osteoclasts, the increase in the histomorphometric and gene expression measures of bone resorption in the presence of high concentrations of estradiol in Orx male-to-female mice was intriguing. We speculate that the actions of estradiol to increase osteoblast proliferation and bone mass in these mice also resulted in a concomitant increase in the secretion of coupling factors by osteoblasts, which increased osteoclastogenesis. While this would be expected to decrease bone mass, it is plausible that the high concentration of estradiol suppressed the resorptive capacity of the osteoclasts, resulting in an uncoupling of bone remodeling with bone formation exceeding bone resorption and leading to increased bone mineral accrual. A similar phenotype is observed in patients with osteoclast-rich osteopetrosis, whereby bone formation is normal or increased in the presence of normal or increased numbers of multinucleated osteoclasts with impaired ruffled-border formation and resorption capacity, thus leading to increased bone formation in the presence of increased markers of resorption.^40^ Confirmation of this hypothesis would require further in-depth investigation including measures of osteoclast function (i.e., actin ring and ruffled border formation) together with an extensive analysis of the coupling process between bone resorption and formation, which is a multifaceted process involving numerous regulators.^41^ The mechanism of estradiol action to increase trabecular bone accrual in the current study by stimulating the formation of primary spongiosa in addition to increasing bone formation in the secondary spongiosa suggests that in trans girls, GAHT should be started as soon as possible after pubertal suppression to maximize the formation of new trabeculae from the growth plate before its fusion upon withdrawal of GnRHa treatment.

While there are very few studies investigating the effects of estradiol treatment in male Orx mice either prepubertally or in early puberty, a dose-dependent increase in the tibial bone ash weight and the levels of calcium and phosphorus as well as an increased metaphyseal and epiphyseal bone area were previously reported in male Orx mice at 3 weeks of age treated with increasing doses of estradiol 1 week post-surgery for 4 weeks.^42^ No effect of estradiol treatment was observed on these parameters in intact male mice. The authors suggest a possible antagonistic effect of testosterone on estradiol actions within bone such that intact males do not respond to estradiol, but in the absence of androgens, estradiol can act on growing male bones in a similar way to that of females.^42^ The mechanism of the anabolic action of estradiol to strikingly increase trabecular bone in the Orx male-to-female mice is unclear but is likely to be mediated, at least in part, by insulin-like growth factor-1 (IGF-1). Previously, estradiol treatment of adult (12-month-old) male mice at the time of orchidectomy prevented Orx- and age-related loss of the trabecular bone density, which was associated with increased circulating IGF-I concentrations.^43^ It was not possible to measure serum IGF-1 concentrations in the current study due to an insufficient volume of serum remaining following sex steroid analyses, and this warrants further investigation.

Although there was no measurable effect of sex, orchidectomy or estradiol treatment on the femoral length, male controls had a decreased growth plate thickness compared to female controls, consistent with the sexual dimorphism of bone. Orchidectomy in pubertal males increased the growth plate height, while estradiol treatment to Orx male-to-females restored the growth plate height to a level that was intermediate between male and female controls 12 weeks post-treatment. Evidence from cartilage-specific *ERα* knockout mice suggests that the mechanism of action for the decrease in growth plate height following estradiol treatment in Orx male-to-female mice is likely to be direct via ERα to decrease chondrocyte proliferation in adulthood.^44^

The marked increase in cortical and trabecular bone accrual following estradiol treatment in the Orx male-to-female mice in the current study contrasts with several reports in adolescent trans girls in whom GAHT was unable to fully restore the BMD and peak bone mass following puberty suppression. Although GAHT following GnRHa treatment in trans girls has been shown to increase the BMAD at both the lumbar spine and femoral neck and the aBMD at the lumbar spine, the BMAD Z scores did not recover to pre-GnRHa treatment scores and remained below zero,^3,4,7^ while levels of serum markers of bone remodeling were decreased.^3,7^ These differences may be ascribed to the dosing regimens of estradiol used, with a recent study suggesting insufficient serum estradiol concentrations as a possible reason for failure of the BMD Z score catch-up despite long-term GAHT.^8^ In contrast to our dosing regimen of GAHT in the current preclinical study, whereby delivery of estradiol via a silastic implant delivered a sustained high concentration of estradiol, often in trans girls, estradiol is administered in gradually increasing doses every 6 months for the first two years of treatment until a maximum of 2 mg/d and a sustained physiological concentration of 100–200 pg/mL serum estradiol is achieved,^45,46^ although exact clinical treatment regimens vary among experts. The marked increase in bone accrual and strength observed in the male-to-female mice in the setting of high serum concentrations of estradiol in the current study suggests that higher doses of estradiol may be needed to restore peak bone mass following puberty suppression in trans girls. Due to the lack of dose response experiments, we cannot directly confirm this hypothesis. Given the complexity of such experiments, we instead chose a single estradiol dose based on preliminary dose-finding experiments. However, our inference that a higher dose of estradiol might overcome bone deficits in trans girls is supported by a recent study by Boogers et al.,^16^ whereby trans girls on GnRHa with very tall height prediction were treated with a higher than standard dose of estradiol (6 mg/d vs. 2 mg/d). After a median of 2.8 years after treatment with estradiol, trans girls treated with the higher dose had a median serum estradiol concentration of 522 pg/mL and a greater increase in height-adjusted BMD Z scores (HAZ-scores) in both the lumbar spine and femoral neck compared to those treated with the standard dose and a median serum estradiol concentration of 126 pg/mL. The HAZ scores in the trans girls treated with the higher dose returned to the pretreatment HAZ scores after two years of GAHT, while those in trans girls receiving the regular dose of estradiol did not. Whether this increase in BMD with the higher estradiol dose is maintained once the patient has reached their expected height and begins receiving the regular dose is not known. Overall, consistent with our rodent model of adolescent male-to-female transition, the existing evidence suggests that skeletal integrity can be preserved if a dose of estradiol is administered that is sufficiently high. However, whether similar doses to those administered in our rodent models can be administered safely to trans girls or what the optimal dose of estradiol would be in trans girls is an important clinical question that requires further study. In this context, potential risks of higher-dose estradiol, e.g., thromboembolic complications, must be taken into consideration; of note, while no overt adverse events were noted with regular monitoring of the mice as per ethics approval, our study was not designed to assess risks of higher-dose estradiol, especially in humans.

The marked increase in cortical and trabecular bone accrual following estradiol treatment in the male-to-female mice suggests that the intraskeletal concentrations of estradiol were increased by the high dose of estradiol administered. In the setting of low serum testosterone concentrations and high serum estradiol concentrations, these data infer that an increase in the intraskeletal concentration of estradiol arose via diffusion into bone from the circulation rather than from the aromatization of testosterone by osteoblasts within bone. Confirming this notion requires the measurement of the intraskeletal concentrations of estradiol. Despite our efforts, we have yet to validate an LCMS method for measuring intraskeletal concentrations of sex steroids, which remains a current line of investigation.

Additional limitations of this study not previously discussed may include the use of orchidectomy rather than GnRHa therapy to reduce sex steroids to castration concentrations, although it is theoretically possible that due to their differential effects on gonadotrophins, these treatments may not have the same bone effects.^47^ The skeletal deficits observed in Orx male mice are broadly consistent with those reported for puberty-suppressed female adolescents and GnRHa-treated male mice.^3,7,30,33^ The marked increase in bone mass, which led to a visible reduction in marrow space, may have adversely affected blood cell production, resulting in anemia. The limited blood volume available for analysis prohibited these measurements. It is also possible that the increased bone mass and decreased marrow space in estradiol-treated male-to-female mice compared to control mice altered the cell populations within bone, thereby potentially confounding the gene expression data. Finally, the small number of samples for the histomorphometry analyses in some of the groups likely decreased the power to detect significant differences. Nevertheless, it is encouraging that the findings from the histomorphometric and gene expression analyses were congruent and aligned with the effect of estradiol treatment on bone structure determined by μCT in the male-to-female mice.

In conclusion, we have shown that the failure to achieve peak trabecular and cortical bone mass accrual in male-to-female mice and the deficit in bone strength compared to control males following pubertal suppression can be rectified by administration of a sustained, high dose of estradiol in late puberty. In fact, the high physiological concentrations of circulating estradiol achieved in the male-to-female mice in the current study were anabolic for bone, markedly increasing trabecular and cortical bone deposition, which translated to an increase in bone strength compared to both male and female controls. The increased bone accretion in the setting of low serum concentrations of testosterone in the male-to-female mice treated with estradiol suggests that the high serum concentrations of estradiol circumvented the requirement for local conversion of testosterone to estradiol by aromatase within bone. For these data to be informative for the preservation of skeletal integrity and strength of trans girls on GnRHa and GAHT, further study is needed comparing lower doses of estradiol to achieve circulating concentrations closer to the mid- and upper ranges of the proestrus cycle in female mice to better reflect the standard and high doses of GAHT prescribed for trans girls.^16^ Importantly, for clinical relevance, these future studies will need to address the possible adverse side effects of estradiol treatment (i.e., long-term cardiovascular risk and venous thromboembolism^48^). Nonetheless, these data are consistent with our hypothesis and imply that it is possible to preserve bone health and prevent long-term fractures in trans girls treated with GnRHa and GAHT if a dose of estradiol that protects the bone without adverse side effects can be identified. Given the lack of information assessing either bone microstructure by high-resolution modalities such as HR-pQCT or fracture prevalence following pubertal suppression and GAHT in trans girls, future studies in this area should be considered a research priority.

## Materials and methods

### Animal husbandry and housing

Fifty male and 10 female C57/BL6J mice at 4 weeks of age were purchased from the Walter and Eliza Hall Institute (Parkville, Australia). Mice were supplied with sterile autoclaved water and standard irradiated mouse chow (BARASTOC Irradiated WEHI Mice Cubes, 1.2% calcium, 0.96% phosphorus, 9% fat, 22% crude protein) *ad libitum*. Mice were housed in a specified pathogen-free facility at 22 °C with a 12-h light/dark cycle in standard cages, 2–5 per cage. The welfare of the mice was monitored on a weekly basis, and all studies were conducted in accordance with accepted standards of humane animal care and performed with the approval of the Austin Health Animal Ethics Committee (A2019/05638) and in accordance with the ARRIVE guidelines.^49^

### Experimental design

To model puberty suppression in adolescent trans girls, male C57BL/6 mice were Orx at 5 weeks of age, representing a time shortly after the onset of puberty in male C57BL/6 mice, which occurs at a median of 30 days as measured by balano-preputial separation.^50^ Male mice were randomly allocated to either sham control or orchidectomy surgery, while all female mice underwent sham control surgery performed under isoflurane anesthesia. At 3 weeks post-surgery, tissues were collected from 10 male sham controls and 10 male Orx mice to serve as pretreatment controls. The remaining male mice were randomly allocated to receive vehicle (control empty 0.2 cm silastic implant) or a 0.2 cm silastic implant containing ~0.85 mg crystalline 17β-estradiol administered intraperitoneally into the abdominal fat pad under isoflurane anesthesia. All female control mice were administered an empty vehicle implant. Mice were pair-fed for the duration of the experiment where the weekly food amount provided was adjusted to match the average weekly food intake of *ad libitum* male mice treated with vehicle. Ten and three days before sacrifice, the mice were injected with 20 mg/kg calcein (Sigma, Australia). Following 12 weeks of treatment, the mice were fasted overnight (14–16 h) and the following morning (8:30 a.m. to 10:30 a.m.) Mice were weighed and anesthetized by intraperitoneal injection of 130 mg/kg ketamine and 10 mg/kg xylazine. Blood was collected via cardiac puncture followed by cervical dislocation, and tissues were collected. No overt adverse events were noted with regular monitoring of the mice as per ethics approval. One femur was fixed in 10% buffered formalin (Sigma, Australia) for 4 days and stored in 70% ethanol for microcomputed tomography (μCT) analysis, after which the femurs were embedded in methylmethacrylate for dynamic histomorphometry.^26^ The remaining femur was wrapped in saline-soaked gauze and stored at -20 °C for 3-point bending analyses. One tibia, including bone marrow, was homogenized in solution D and stored at -80°C for RNA preparation as described previously.^51^ All analyses were performed in a blinded fashion.

### Sex steroid analyses

To accurately determine the low serum concentrations of sex steroids previously reported in control mice, the serum concentrations of testosterone and estradiol were determined using an ultrasensitive estrogen-specific derivatization liquid chromatography tandem mass spectrometry (LCMS) method as described previously.^19^ The limit of detection (LOD) was 0.01 ng/mL for testosterone and 0.5 pg/mL for estradiol.

### Bone analyses

Right femoral length was measured using a digital Vernier caliper (Etalon, Switzerland). Bone microarchitecture was evaluated by μCT scanning (Skyscan 1272, Bruker, Belgium). Scans were performed with an X-ray tube potential of 45 kV and 200 μA, a resolution of 5.2 microns, and a rotation step of 0.4 degrees with a 0.25 mm aluminum filter. Reconstruction of transverse μCT slices was generated using NRecon (v2.0.4.5, Skyscan, Belgium), and images were reconstructed and thresholded using a specimen-specific threshold based on the Otsu method.^52^ Cortical parameters were determined in a 0.68 mm region of cortical bone, equivalent to 130 CT slices, centered on a CT slice located at half of the total femoral length from the distal end. The distal femoral growth plate height in the midcoronal plane was automatically segmented and measured by a 2D implementation of the sphere-fitting local thickness method.^53^ The primary spongiosa of the distal femoral metaphysis was automatically segmented and evaluated in terms of trabecular parameters in the midcoronal plane in a region of interest extending 265 μm from the growth plate. Trabecular bone parameters were determined in the secondary spongiosa of the distal femoral metaphysis, delineated automatically and manually, and evaluated in the volume of interest commencing 265 μm below the growth plate extending 1.5 mm. Cortical and trabecular parameters were calculated using a direct 3-dimensional approach using CTAn software (v1.18.4.0; Skyscan). Changes in tissue mineral content were quantified using multilevel Otsu thresholding as described by Walker et al. ^54^ Bone volume as a percentage of total metaphyseal volume was measured at low-, mid- and high-density thresholds. The threshold ranges for the three density levels of bone measured were low density (0.624–1.230 g/cm^3^ CaHA), mid-density (>1.230–1.789 g/cm^3^ CaHA), and high density (>1.789 g/cm^3^ CaHA). The lowest-density quartile containing nonbone material was discarded.

Dynamic histomorphometry was performed in the same region of the secondary spongiosa of the distal femoral metaphysis as for the micro-CT analyses.^55^ Five-micrometer-thick sagittal sections were stained with tartrate-resistant acid phosphatase for Oc.N and Oc.S calculations. Unstained sections were used for evaluation of calcein fluorescence to determine MS/BS, MAR and BFR.^56^ All analyses were performed using the Osteomeasure system (v4.1.0.1; SciMeasure Analytical Systems, GA, USA).

### 3-point bending

To evaluate mechanical bone strength and resistance to bending, 3-point bending was conducted at the mid-diaphysis of the left femur using a materials testing machine (Zwick Z005, Germany) equipped with a 5 kN load cell and built-in displacement control. Tests were performed at a rate of 0.5 mm/s, preload of 1 N, and span length of 7 mm. The femur was positioned with the anterior surface upwards.

Force‒displacement data were analyzed using Microsoft Excel version 16.31 to determine both the structural and material properties of bone. Stiffness was defined as the linear slope of the load‒displacement curve, and the yield point was defined as the point where the curve crossed 90% of the stiffness slope. Centroid distance and minimum moment of inertia in the y-plane were derived from μCT scans (CTAn, Bruker, Belgium).

### Real-time quantitative PCR (Q-PCR)

Total RNA was extracted from whole tibia, and cDNA synthesis was performed as described previously.^26^ To determine mRNA levels of genes of interest, quantitative (Q)-PCR was performed in duplicate using Applied Biosystems TaqMan gene expression assays (Supplementary Table) and a QuantStudio^TM^ 3 Real-Time PCR System (Thermo Fisher Scientific, MA, USA). Absolute expression was calculated using the ∆∆C_T_ method, with values for the gene of interest normalized to a housekeeping gene validated for stability of expression in the current study and previously (*Eef2* for 3 weeks post-surgery,^57^ *Hprt1* for 12 weeks post-treatment;^58^ Table S1) and expressed relative to a reference sample.

### Statistical analysis

All statistical tests were performed using GraphPad Prism 9.0 for MacOS. The effect of orchidectomy on bone parameters at 3 weeks post-surgery was determined with an unpaired two-tailed *t* test or a Mann‒Whitney test for data that were normally or not normally distributed, respectively. The effect of estradiol treatment on Orx males at 12 weeks post-treatment compared to male and female controls was identified by one-way ANOVA with specific differences identified by Tukey’s *post hoc* test for normally distributed data or a Kruskal‒Wallis and Dunn’s multiple comparisons test for nonnormal data. A value of *P* < 0.05 was considered significant.

### Supplementary information

Supplementary Table 1 and Supplementary Figure 1

## Data Availability

The data analyzed to support the findings in this study are available from the corresponding author upon request.
