# Supplementary material for: Estradiol increases cortical and trabecular bone accrual and bone strength in an adolescent male-to-female mouse model of gender-affirming hormone therapy
Source: Bone Res. 2024 Jan 11;12:1. doi: 10.1038/s41413-023-00308-2 (PMC10784310; doi:10.1038/s41413-023-00308-2)
Supplement: Supplementary file 1 — Supplementary Table 1 and Supplementary Figure 1 [file 41413_2023_308_MOESM1_ESM.docx]

**Supplementary Table 1:** Taqman Gene Expression Assays used for Quantitative Real Time PCR (Q-PCR).

| Gene Name | Gene Symbol | Entrez Gene ID | Taqman Assay ID |
| --- | --- | --- | --- |
| Tartrate resistant acid phosphatase | Acp5 | 11433 | Mm00475698_m1 |
| Cathepsin K | Ctsk | 13038 | Mm00484036_m1 |
| Collagen, type 1, alpha 1 | Col1a1 | 12842 | Mm00801666_g1 |
| Dendrocyte expressed seven transmembrane protein | Dc-stamp | 75766 | Mm04209236_m1 |
| Bone gamma-carboxyglutamate protein 2 | Bglap | 12097 | Custom made probe* |
| Eukaryotic translation elongation factor 2 | Eef2 | 13629 | Mm00833287_g1 |
| Hypoxanthine phosphoribosyltransferase 1 | Hprt1 | 15452 | Mm00446968_m1 |

* Huang, J. C. *et al.* PTH differentially regulates expression of RANKL and OPG. *J Bone Miner Res* **19**, 235-244 (2004).


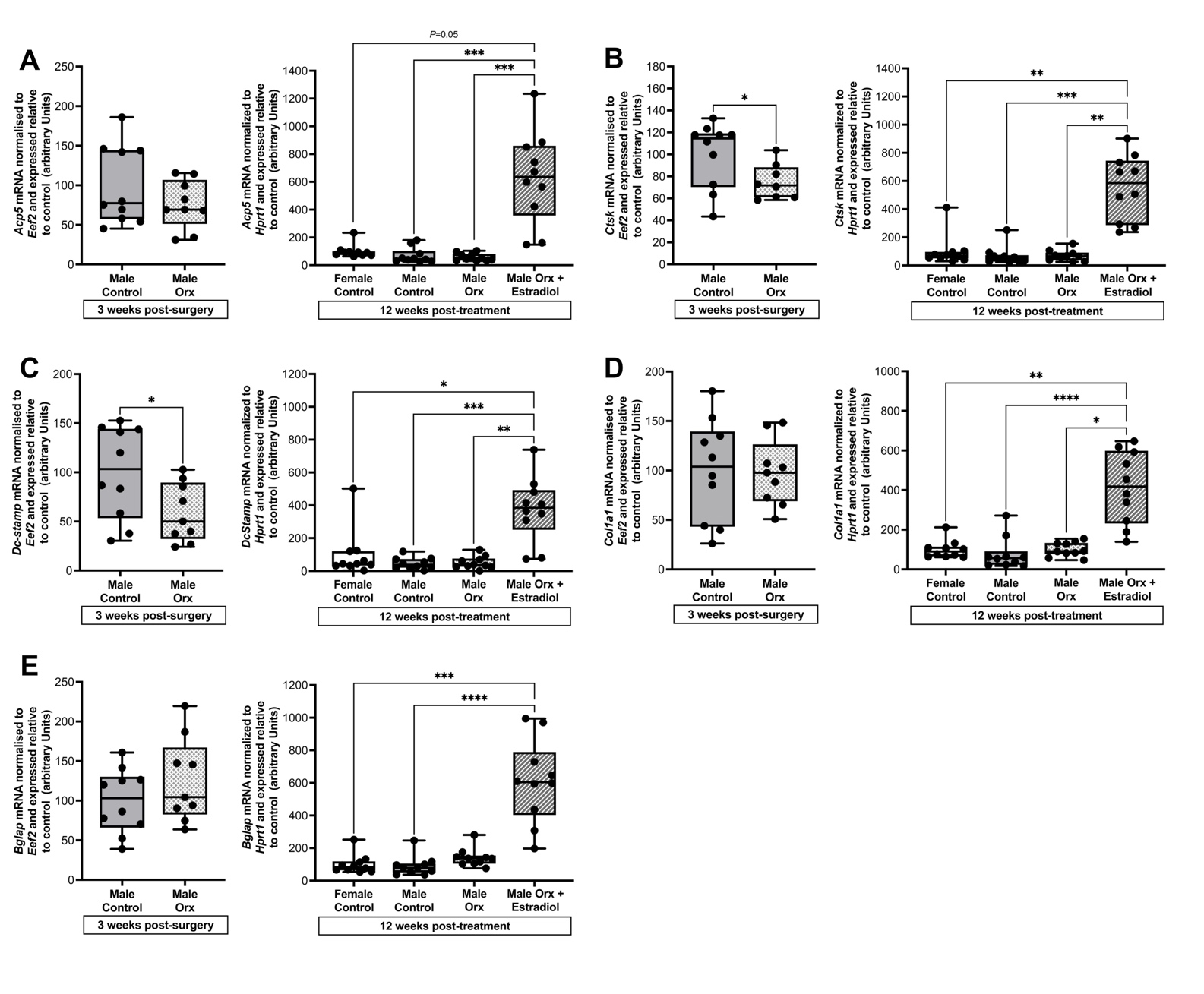
**Supplementary Figure 1: The expression of genes involved in bone remodelling are markedly upregulated in estradiol treated Ovx male-to-female mice.**

**A)** Acp5, **B)** Ctsk, **C)** Dc-Stamp, **D)** Col1a1 and **E)** Bglap gene expression in whole bone of male controls (n=10) and Orx males (n=10) 3 weeks post-surgery (8 weeks of age), and in female controls (n=10), male controls (n=10), Orx males (n=9) and Orx males administered estradiol (male Orx + Estradiol)(n=10) at 3 weeks post-surgery (8 weeks of age) for 12 weeks (20 weeks of age). mRNA levels are normalized to a housekeeping gene and expressed relative to male controls at 3 weeks post-surgery or to female controls at 12 weeks post-treatment. **P*<0.05, ***P*<0.01, ****P*<0.0005, *****P*<0.0001.
